# Sexual function and quality of life following retropubic TVT and single-incision sling in women with stress urinary incontinence: results of a prospective study

**DOI:** 10.1007/s00404-012-2669-8

**Published:** 2012-12-15

**Authors:** Gert Naumann, Joscha Steetskamp, Mira Meyer, Rosa Laterza, Christine Skala, Stefan Albrich, Heinz Koelbl

**Affiliations:** Department of Obstetrics and Gynecology, University Medical Center, Johannes Gutenberg-University Mainz, Langenbeckstrasse 1, 55131 Mainz, Germany

**Keywords:** Female Sexual Function Index, Sexual function, TVT, Single incision sling, Stress, Urinary incontinence, Success rate

## Abstract

**Purpose:**

The objective of this prospective cohort study was to compare effectiveness, morbidity, quality of life (QoL) and sexual function in women treated with tension-free vaginal tape (TVT) versus single-incision sling (SIS) in the treatment of female stress urinary incontinence (SUI).

**Methods:**

Retropubic TVT sling or SIS was implanted in local anesthesia and patients were followed post-operatively for 6 months. Evaluation was performed to assess post-operative rate of continence, complications, changes in sexual function and patient reported quality of life. Female sexual function was evaluated before and after sling procedure using Female Sexual Function Index (FSFI) in sexually active patients.

**Results:**

From January 2009 to December 2011, 150 patients were enrolled and underwent a procedure to implant the retropubic TVT (*n* = 75) or the MiniArc^®^ and Ajust^®^ SIS (*n* = 75). Overall, 93.3 % of the patients who successfully received SIS demonstrated total restoration (84 %) or improvement of continence (9.3 %) at the 6 month post-operative study visit. In TVT group we found 88 % total continence and 6.7 % improvement, respectively. Improvements were seen in the QoL scores related to global bladder feeling (89.3 %) in SIS group and 96 % for TVT. Post-operative FSFI score improves significantly and were comparable in both groups (SIS pre-operative 24.30 ± 4.56 to 27.22 ± 4.66 (*P* < 0.001) post-operative; TVT 24.63 ± 6.62 to 28.47 ± 4.41, respectively).

**Conclusions:**

The SIS procedure appears to be as effective in improving incontinence-related quality of life and sexual function as the TVT through 6 months of post-operative follow-up. No differences in complications and sexual function were demonstrated between the groups.

## Introduction

Urinary incontinence is a prevalent disorder reported by up to 25 % in women and is highly correlated with impairment of quality of life (QoL) and sexual dysfunction [1, 2]. Curing of incontinence without generating new side effects (e.g., pain, mesh erosion) may lead to an improvement of sexual function. Surgical and post-operative complications such as de novo urgency, pain or dyspareunia negatively affects sexual function.

Today the minimally invasive mid-urethral slings have become a standard surgical procedure for surgical treatment of stress urinary incontinence (SUI) in women and has resulted in a rapid increase in the number of surgeries performed throughout the last 15 years. Single-incision slings (SIS) with anchor fixation in the musculus obturatorius internus fascia or membrane, such as the MiniArc^®^ and Ajust^®^ sling, represent the most recent advancement in the treatment of SUI. This minimally invasive procedure leads to less complications with comparable results to conventional slings [3]. Only few long-term data are available; 2-year follow-up data from the MiniArc and Ajust system demonstrate continence for 82–93 % [4–7] of all patients; no severe side effects were seen.

The effect of suburethral sling on sexual functioning has been studied, but the results are still inhomogen. Current literature shows inconsistent data with some studies reporting improvement [8, 9], some authors reporting deterioration [10–12] and other noting no change of sexual function symptoms [13–15].

The aim of our study was first to prospectively evaluate the effect of surgical procedure for SUI on sexual function in sexually active patients as well as QoL prospectively; second to compare the retropubic approach with TVT sling with single-incision mid-urethral sling procedure MiniArc and Ajust on this regard. Different approaches of sling insertion may lead to a different outcome in sexual function. Minimally invasive access with the avoidance of relevant anatomical structures, less synthetic material and missing blind passage of SIS should lead to an improvement of incontincence related disorders as well as sexual function while preventing conventional surgery related side effects. Restrictively, using the transobturator approach of SIS could adversely affect sexual function more than retropubic way of TVT due to the closer contact to the dorsal nerve of the clitoris [16].

## Materials and methods

In this prospective cohort study, we enrolled patients to a suburethral sling for female SUI between January 2009 and December 2011 in our University Department of Obstetrics and Gynecology. In total, 150 patients were included, divided into groups for correction of SUI: 75 underwent insertion of retropubic sling TVT and 75 of transobturator SIS MiniArc^®^ and Ajust^®^. Randomisation of patients for the use of TVT or SIS sling was done in accordance with the clinical guidelines of the hospital. Ethical permission was obtained from the regional ethics committee at University Mainz, Germany and all the patients provided written informed consent for participation.

Patient recruitment with pre-operative urodynamics and urogynecologic evaluation was in accordance with to International Continence Society (ICS) guidelines [17].

All the patients showed pure SUI and normotonic urethra (maximum urethral closure pressure at rest of more than 20 cm of water in urethral profilometry using an external fluid-filled catheter).

The retropubic TVT^®^, MiniArc^®^ and Ajust^®^ sling procedures were performed as per the manufacturer’s instructions provided in the instructions for use. TVT sling was performed as originally described by Ulmsten from vaginally to above with the complete tension-free placement of sling under the midurethra. Both transobturator SIS systems are comparable and use special anchors which fixate the sling on both pelvic sides. Procedures were initiated with a 1 cm mid-urethral vaginal incision and preparation of bilateral para-urethral tunnels to the posterior margin of the inferior pubic ramus. Surgeons used a special thin introducer, loaded with self-fixating anchors, to pivot around the ischiopubic ramus and insert the anchor through the obturator internus muscle and obturator membrane. In all the procedures for TVT and SIS the performance of cystoscopy were done for the exclusion of bladder lesions. All sling implantation was performed under local anesthesia. The Foley catheter was removed within 24 h after surgery.

Exclusion criteria used were: urge incontinence, mixed urinary incontinence with dominant urge component, intrinsic sphincter deficiency, neurogenic bladder (multiple sclerosis, meningomyelocele, spinal cord injury), symptomatic pelvic organ prolapse, previous incontinence and prolapse surgery, severe mental illness, severe physical handicap, poor knowledge of German language and refusal to consent. To reduce age-related bias, patients over 75 years were excluded.

All women were sexually active; participants who had no sexual activity within 3 months prior to surgery were excluded from the study.

Patients were evaluated pre-operatively and 6 months post-operatively for Female Sexual Function Index (FSFI), the Patient Perception of Intensity Urgency Scale (PPIUS) and the Patient Global Impression of Improvement (PGI-I). Effect of sling surgery on sexual function and the assessment of sexual function, continence and QoL were investigated prospectively.

Assessing of post-operative rate of continence-related QoL and changes in sexual function were the primary objectives of this study. Continence was evaluated by a standardized cough stress test (CST) with a bladder volume of approximately 300 ml (confirmed by ultrasound, GE Voluson-e^®^ System); pre- and post-operative FSF scores were evaluated to determine the changes in sexual function. Later, we included the evaluation of intra-, peri- and post-operative complications (bleeding >200 ml, bladder lesions, erosions, perforations, penetrations, protrusions, hematomas and bladder outlet obstructions) and patient QoL.

All women undergoing the TVT or SIS sling procedure were instructed to complete the FSFI before and after surgery. The FSFI is a detailed 19-item anonymous questionnaire including sexual desire (score range 2–10), arousal (score range 0–20), lubrication (score range 0–20), orgasm (score range 0–15), satisfaction (score range 2–15), and pain during sexual intercourse (score range 0–15), as described by Rosen et al. [18]. A validated German language version of the FSFI was used [19]. Total score was obtained by adding the six domain scores and was calculated multiplying the sum by the domain. Factors were 0.6 for desire, 0.3 for arousal and lubrication, 0.4 for orgasm, satisfaction, and pain. Therefore, total FSFI score range was 2–36 (see Table 1).

**Table 1:** FSFI domain scores and full scale score (18)

Domain	Questions	Score range	Factor	Minimum score	Maximum score
Desire	1, 2	1–5	0.6	1.2	6.0
Arousal	3, 4, 5, 6	0–5	0.3	0	6.0
Lubrication	7, 8, 9, 10	0–5	0.3	0	6.0
Orgasm	11, 12, 13	0–5	0.4	0	6.0
Satisfaction	14, 15, 16	0 (or 1)–5	0.4	0.8	6.0
Pain	17, 18, 19	0–5	0.4	0	6.0
Full scale score range				2.0	36

Patients also completed the PGI-I, a validated, seven-item questionnaire used to assess improvement with therapy (very much better, much better, a little better, no change, a little worse, much worse and very worse) [20].

Urgency was evaluated using the PPIUS which scored the intensity of urgency at each void with a 5 point scale [21]. Questionnaires were completed by the patients prior to clinical examination for baseline and follow-up assessment.

The data were analyzed at the end of the study using the Excel program (Microsoft Office Excel 2011). Statistical analysis was performed using the Statistical Package For Social Sciences (SPSS^®^, Version 17, Chicago, IL). Normality testing (Kolmogorow-Smirnow test) was performed to determine if data were sampled from a normal distribution. The *t* test and Mann–Whitney *U* test were performed to compare continuous parametric and non-parametric variables, respectively. The proportion of categorical variables was analyzed for statistical significance using *chi*-square test. Statistical significance was considered to have been reached when *P* value was less than 0.05.

## Results

From January 2009 to December 2011, 150 patients with SUI were enrolled in this study and underwent a surgical procedure to receive the retropubic sling TVT^®^ (*n* = 75) or single incision sling MiniArc^®^ (*n* = 57) and Ajust^®^ (*n* = 18) SIS. All the patients completed the scheduled post-operative 6-month follow-up visit. Demographic and baseline characteristics are shown in Table 2. There were no statistically significant differences between both groups in terms of demographics, pre-operative QoL measures and urodynamic results.

**Table 2:** Pre-operative characteristics

	SIS group (*n* = 75)	TVT group (*n* = 75)	*P* value
Age (years)^a^	55.3 (41–72)	54.1 (32–70)	0.415
Parity^a^	2 (1–4)	2 (0–4)	0.813
Menopause (*n*)	46 (61.3 %)	39 (52 %)	0.249
Hysterectomy (*n*)	14 (18.7 %)	17 (22.7 %)	0.539
Hospital stay (days)^a^	2.2 (1–3)	2.7 (1–4)	0.422

*SIS* Single-incision sling, *TVT* retropubic tension-free vaginal tape

^a^Median and range

Perioperative data and outcomes from 6 month visit are compared in Table 3. None of the patients had concomitant surgeries and sling implantation was done under intravenous sedation and local anesthesia in all patients. The mean operating time and complications were comparable in both groups. All 150 placement procedures were successful, no complications of bleeding >200 ml, bladder lesions, perforations, penetrations, protrusions, hematomas or bladder outlet obstructions were reported during the study. Operating time and duration of in-patient treatment were comparable in both groups, in previous studies, country specific reimbursement schedules had influenced the length of hospitalization for TVT and SIS insertion.

**Table 3:** Perioperative data and follow-up outcomes

	SIS group (*n* = 75)	TVT group (*n* = 75)	*P* value
Operative time (min)^a^	18.3 (11–24)	19.4 (12–26)	NS
Intraoperative complication	0	0	NS
Bladder lesion	0	0	NS
Blood loss > 200 ml	0	0	NS
Haematoma	0	1	NS
Nerval or bowel injury	0	0	NS
Urgency			
Day after procedure	1	4	<0.05
After hospitalization	1	3	<0.05
Voiding dysfunction (RU >100 ml)			
Day after procedure	0	6	<0.001
After hospitalization	0	1	<0.05
Vaginal erosion	1	1	NS
Tape releasing	2	2	NS

*SIS* Single-incision sling, *TVT* retropubic tension-free vaginal tape

^a^Including time of intraoperative urethrocystoscopy

Intraoperative and post-operative pain scores were consistently low with no cases of groin pain reported post-operatively and after 6 months of follow-up in both groups.

The overall rate of vaginal sling erosion after surgery was low (1.3 %) and not significantly different between the two groups and erosions into the bladder or urethra were not noted.

### Impact on incontinence-related quality of life function

Results for continence are listed in Table 4. At the 6-month study visit, 63 (84 %) of the 75 patients who received the SIS-sling demonstrated total restoration with negative CST or improvement of continence (*n* = 7; 9.3 %). Five patients (6.7 %) demonstrated no SUI improvement over baseline. Subjective continence assessment demonstrated that 67 (89.3 %) of all 75 patients showed high satisfaction with subjective continence after surgery. There was no difference of type of SIS for objective or subjective results. Seven months after SIS insertion, one patient with no SUI improvement received another incontinence procedure with Burch colposuspension after which complete continence was restored. In the TVT group we found *n* = 66 (88 %) cases of fully continence with *n* = 5 (6.7 %) improvement and *n* = 4 (5.3 %) cases of failure; 72 patients (96 %) showed high satisfaction concerning post-operative continence.

**Table 4:** Evaluation of objective and subjective continence rate and PGI-I in TVT group and SIS group 6 months post-operatively

	SIS group (*n* = 75) (%)	TVT group (*n* = 75) (%)
Objective continence	63 (84)	66 (88)
Objective improvement	7 (9,3)	5 (6.7)
Objective failure	5 (6,7)	4 (5.3)
Subjective continence and satisfaction	67 (89.3)	72 (96)
Patient impression of global improvement (PGI-I)		
1: very much better	59 (78.7)	57 (76)
2: much better	8 (10.7)	12 (16)
3: a little better	3 (4.0)	2 (2.7)
4: no improvement	4 (5.3)	3 (4.0)
5: a little worse	1 (1.3)	1 (1.3)
6: much worse	0 (0)	0 (0)
7: very much worse	0 (0)	0 (0)
Success*	67 (89.4)	69 (92)
Comparison of mean PGI-I scores		
*t* test for unpaired samples	*p* = *0.925*	

*SIS* Single-incision sling, *TVT* retropubic tension-free vaginal tape, *PGI-I* Patient impression of global improvement, Success* ‘very much better’ and‘ much better’

PGI-I questionnaire data were collected and reported at the month 6 study visit. Of the 150 patients 90.7 % (*n* = 136) showed a successful outcome with “very much better” (*n* = 16) or “much better” (*n* = 20), with no statistically significant difference between the two groups (TVT 92 % versus SIS 89.4 %, *p* = 0.925) (Table 4).

Post-operatively, patients who received TVT were significantly more likely to develop voiding dysfunction and urgency than those who received a SIS. Data available from baseline through 6 months of post-operative follow-up showed significant more urgency in TVT group compared with SIS. Sixty-nine (90.6 %) of the 75 patients who received a SIS reported PPIUS scores of 0 or 1; indicating the majority of patients feeling no urgency or being able to postpone voiding as long as necessary without fear of wetting at each void, compared to *n* = 61 (81.3 %) in TVT group (Table 5).

**Table 5:** Evaluation of PPIUS in TVT group and SIS group 6 months post-operatively

PPIUS Rank	SIS group (*n* = 75) (%)	TVT group (*n* = 75) (%)	*P* value
0: no urgency	61 (81.3)	39 (52)	
1: mild urgency	7 (9.3)	22 (29.4)	
2: moderate urgency	5 (6.7)	12 (16)	
3: severe urgency	2 (2.7)	1 (1.3)	
4: urge incontinence	0 (0)	1 (1.3)	
Mean PPIUS score (SD)	0.31 (0.716)	0.71 (0.882)	0.003

*SIS* Single-incision sling, *TVT* retropubic tension-free vaginal tape, *PPIUS* patient perception of intensity of urgency scale

Based on clinical assessment, PPIUS scores of 2 or greater were considered abnormal and represented cases of de novo (new onset) urgency. Five patients in SIS group experienced moderate urgency (PPIUS score of 2) after the 6-month-follow-up. Two other patients reported severe urgency. All seven cases were treated with anticholinergic medications and treatment was ongoing at the end of study participation. In TVT group significantly more cases of moderate (*n* = 12) and severe (*n* = 2) urgency were detected (*p* = 0.003). These patients also needed anticholinergic medication in the follow-up.

The majority of the patients in both groups (96 %; 144/150) having PVRs of <100 ml at 1 day post-surgery, all six women with PVR >100 ml belong to the TVT group (*p* < 0.005). Two women in each group needed operative release of their sling.

### Impact on sexual function

All pre-operative sexually active women reported resumption of sexual activity after sling surgery. Evaluation of sexual function pre-operatively and after 6 month of post-operative follow-up, FSFI scores showed an improvement in all domains for the majority of patients. A total of 83 (53.3 %) showed the improvement in total FSFI scores, whereas 10 patients (6.7 %) showed deteriotation, and 57 patients (38 %) had no changes for FSFI questionaire. These changes of sexual function were comparable in both groups (TVT *n* = 42 improvement and *n* = 4 deteriotation; respectively SIS *n* = 41 improvement and *n* = 6 deteriotation) without any significant differences.

Comparison of mean pre- and post-operative mean FSFI scores revealed a significant improvement of the total FSFI score in both groups in the total score and in all domains at 6 months without significant differences between TVT and SIS group. Table 6 demonstrates the mean difference between the baseline and month 6 FSFI scores for both groups. At month 6, the mean follow-up FSFI total score in SIS group improved from baseline 24.30 ± 4.56–27.22 ± 4.59 (*P* < 0.001), in contrast to the TVT group from baseline 24.63 ± 6.62–28.47 ± 4.41 (*P* < 0.001).

**Table 6:** FSFI domains score of SIS group and TVT group pre-operatively and 6 months follow-up

	Desire	Arousal	Lubrication	Orgasm	Satisfaction	Pain	Total
FSFI scores SIS group (*n* = 75)							
Pre-operatively	3.18 ± 0.86	3.81 ± 1.16	4.28 ± 1.36	4.00 ± 1.12	4.72 ± 1.17	4.31 ± 1.25	24.30 ± 4.56
Post-operatively	3.66 ± 0.80	4.37 ± 0.96	4.49 ± 1.33	4.41 ± 0.97	5.27 ± 0.83	5.06 ± 0.99	27.22 ± 4.59
*P* value	<0.001	<0.001	0.009	<0.001	<0.001	<0.001	<0.001
FSFI scores TVT group (*n* = 75)							
Pre-operatively	3.22 ± 0.98	3.94 ± 1.30	4.48 ± 1.45	4.25 ± 1.38	4.58 ± 1.50	4.16 ± 1.42	24.63 ± 6.62
Post-operatively	3.70 ± 0.84	4.46 ± 0.94	5.00 ± 1.12	4.76 ± 1.00	5.29 ± 0.87	5.30 ± 1.00	28.47 ± 4.41
*P* value	<0.001	<0.001	0.003	<0.001	<0.001	<0.001	<0.001
Comparison of FSFI scores post-operatively for TVT and SIS group							
SIS group	3.66 ± 0.80	4.37 ± 0.96	4.49 ± 1.33	4.41 ± 0.97	5.27 ± 0.83	5.06 ± 0.99	27.22 ± 4.59
TVT group	3.70 ± 0.84	4.46 ± 0.94	5.00 ± 1.12	4.76 ± 1.00	5.29 ± 0.87	5.30 ± 1.00	28.47 ± 4.41
*P* value	0.721	0.589	0.012	0.030	0.939	0.143	0.093

*t* test for independent samples

*SIS* Single-incision sling, *TVT* retropubic tension-free vaginal tape

Statistically significant increases in post-operative subdomains sexual desire, arousal, lubrication, orgasm, satisfaction and pain also were observed in both groups.

Comparison of post-operative results between TVT and SIS group in FSFI total and subdomain scores showed no significant differences; only the subdomains, orgasm and lubrication showed an advantage for TVT sling (Table 6).

Only one patient in SIS group was suffering from severe dyspareunia due to sling erosion in the area of colpotomy with hispareunia of the husband. During a second procedure in local anesthesia the exposed material was resected and the vaginal wall was closed; dyspareunia resolved. One patient in TVT group also needed a resection of sling material due to vaginal erosion with moderate pain.

## Discussion

In this prospective study of sexually active women with SUI QoL, urinary continence and sexual function significantly improved after different mid-urethral sling procedures. The insertion of mid-urethral tape has become common practice for the surgical treatment of female SUI. Most long-time experiences over years exists for the retropubic TVT sling system. To avoid complications due to blind needle passage through the retropubic space (TVT sling) or transobturator foramen (TOT sling), new SIS systems were introduced. These slings aim to obtain the same suburethral support with less invasivity by anchoring the two arms in the obturator fascia while avoiding the passage through the adductor muscles.

Clinical reports suggest that SIS is associated with fewer post-operative complications than the TVT. Two-year follow-up data of the MiniArc^®^ system demonstrated continence in 82–93 % of treated patients [4, 5] and 12–24 months of follow up in patients treated with Ajust^®^ showed continence up to 83 % of patients with no severe side effects [6, 7].

In our study both sling systems provide high continence rates without major complications: 93.3 % in SIS group and 94.7 % in TVT group with total continence or improvement support equality of data. PGI-I success rates of 92 % TVT versus 89.4 % in SIS confirm these results. A 6-month follow-up period is very short for the comparison of objective outcome measures to reveal the benefits of different sling systems.

De novo urge symptoms have been a problem with the traditional incontinence operations. Data available from baseline through month 6 observation showed that the majority of all sling patients (86 %) felt no urgency or were able to postpone voiding as long as necessary without fear of wetting at each void. In the TVT group we found significantly more cases of de novo urgency (*n* = 14, 18.7 %) in contrast to *n* = 7 (9.3 %) in SIS group (*p* = 0.003). Corresponding findings on the onset of urge symptoms after TVT surgery have been reported in the literature [22]. Less severe post-operative de novo urgency seen in this study in SIS group was similar to those seen in other patients following transobturator mid-urethral sling placement and was also within range of de novo urgency reported in other SIS Sling studies [3–7].

Sexual dysfunction is more prevalent in women with urinary incontinence or lower urinary tract symptoms than in a general, healthy female population, and is highly prevalent in women suffering from pelvic floor disorders (10–56 %) [23].

Recent publications evaluating the impact of urinary incontinence using the FSFI and PISQ have reported conflicting results with some reporting detoriation [10, 11], or no changing of symptoms [12–15].

The effect of surgery for SUI on sexual function has been controversial. Study design factors including heterogenous patient groups with varying age, hormonal status, neurological and anatomical factors; retrospective design; heterogeneity of urogynecological diagnosis with the coexistence of pelvic organ prolapse; and differences in the evaluated outcome measures have reduced scientific conclusion on this issue.

Results of female sexual function after correction of incontinence by the TVT have been reported inconsistently. Brubaker et al. [8] showed an improvement of sexual function, Rogers et al. [10] found declined sexual function as measured by PISQ in women after TVT despite the improvement of urinary incontinence at 3–6 months.

Most studies evaluating the effect after retropubic sling placement showed not only unchanged sexual function [15] with controversal details in the improvement of symptoms due to solution of urinary leakage but also deterioration due to vaginal scaring, tight insertion of the sling or sling erosion with dyspareunia and partner discomfort.

In our study FSFI total and domain sub-scores in TVT group demonstrate a significant improvement in sexual function, only four patients had deterioation of sexuality after surgery.

Development of the transobturator route for suburethral sling while avoiding the retropubic space led to decreases in complication with comparable cure rates and QoL improvement [24]. Recent studies of the transobturator approach also showed positive effects on sexual function. Studies comparing retropubic and transobturator sling approaches that assessed the improvement of post-operative sexual function [9, 25] noted more pain during intercourse in TOT [26].

Elzevier et al. [27] described a positive effect on sexual function after outside-in (TOT) and inside-out (TVT-O) procedure due to continence during intercourse. More pain during intercourse because of vaginal narrowing was seen significantly more often in the TOT procedure group. Abdel-Fattah et al. [28] demonstrated an improvement in 94 % after 12 months using TVT-O and TOT, and deteriotation in 4.3 %.

First data for sexual function in women with SIS were presented by Pickens et al. [4] in a study with 108 patients recieving MiniArc after showing no change after a follow-up of 12 months. Information was limited due to missing pre-operative FSFI measurement. In another ongoing study from our group we found in 73 patients a significant improvement of sexual function 6 months after SIS insertion using FSFI. Only six patients showed deterioration of sexual function after sling procedure.

There have been no studies which evaluated the changes of sexual function in SISs in comparison to other sling systems like retropubic TVT. In our present study, patients in SIS and TVT group showed comparable significant improvement of sexual function after SIS procedure as evaluated by FSFI. At a 6-month-follow-up the mean FSFI total score improved from baseline 24.30 ± 4.56 to 27.22 ± 4.59 (*P* < 0.001) in SIS group, respectively, 24.63 ± 6.62–28.47 ± 4.41 (*P* < 0.001) in TVT group. All subdomains were changed positively with statistical power. We could not find real differences in pre-operatively and post-operatively FSFI between both groups. There was a statistical difference in subdomain orgasm and lubrication with an advantage to TVT group: for detailed analysis of this possible difference there is a need for bigger sample size.

As a more detailed subgroup analysis would need a bigger sample size and a longer follow-up, we abstained from generating more sub-groups than the described ones. Future research should perhaps include a sample size allowing a more sophisticated subgroup analysis.

## Conclusion

Current evidence of pelvic floor surgery on sexual function is conflicting. Many studies dealing with the changes of sexual function after incontinence surgery show varying results.

Single-incision slings seem to be effective and safe in the operative treatment of female SUI. Six months of follow up data show comparable continence rates for SIS and TVT without severe side effects or complications. Evaluation in QoL shows the improvement after surgery. Focusing on sexual function, we could demonstrate significant improvement due to TVT and SIS procedure. In our study, both sling systems are equally effective in changing continence and sexual function.

Our study warranted several qualifications. To our knowledge, it is the first prospective comparative evaluation in sexual function for a SIS and TVT sling for treatment of SUI with FSFI pre-operatively and after 6 months of follow-up. Despite other studies showing no change or deterioration of sexual function after urogynecological surgery, our study supports a positive effect of SIS and TVT on burden of incontinence and sexual dysfunction.

Limitations of this study include a short follow-up with only 6 months for evaluation of post-operative incontinence. Furthermore, a 6-month follow-up may be too short to test for sexual function improvement. The limited number of patients does not allow the detection of changes in sexual function for patients who do not reach full post-operative continence to evaluate urine loss during intercourse. To compare influence of different slings on changes of sexual function there is need for more subjects in randomized design. The FSFI questionaire does not include questions to explore the relationship between urinary incontinence and sexual dysfunction and there are no clear information about incontinence during intercourse. As the frequency of sexual intercourse turns out to be reduced and FSFI testing only explores the last 4 weeks, there might be a “diagnostic gap” concerning sexual function in the elderly. Female sexual dysfunction is very complex field and not only caused by pelvic floor disorders.

In conclusion, we found the improvement in incontinence and sexual function after single-incision implacement for SUI similarly to TVT.

Establishing new sling modifications such as SIS requires not only evaluation of continence; detection of incontincence-related QoL and sexual function should also be involved in randomized clinical trials.

## Abbreviations

BMI: Body mass index
CST: Cough stress test
FSD: Female sexual dysfunction
FSFI: Female sexual function index
PGI-I: Patient global impression of improvement
PISQ-12: Pelvic Organ Prolapse/Urinary Incontinence Sexual Questionnaire
PPIUS: Patient perception of intensity of urgency scale
PVR: Post-void residual
QOL: Quality of life
SIS: Single-incision sling
SUI: Stress urinary incontinence
TOT: Transobturator tape
TVT: Tension-free vaginal tape
UUI: Urgency urinary incontinence
